# Outcomes among children and adults at risk of severe dengue in Sri Lanka: Opportunity for outpatient case management in countries with high disease burden

**DOI:** 10.1371/journal.pntd.0010091

**Published:** 2021-12-28

**Authors:** Champica K. Bodinayake, Ajith DeS Nagahawatte, Vasantha Devasiri, Niroshana J. Dahanayake, Gaya B. Wijayaratne, Nayani P. Weerasinghe, Madureka Premamali, Tianchen Sheng, Bradly P. Nicholson, Harshanie A. Ubeysekera, Ruvini MP Kurukulasooriya, Aruna D. de Silva, Truls Østbye, Christopher W. Woods, L Gayani Tillekeratne

**Affiliations:** 1 Department of Medicine, Faculty of Medicine, University of Ruhuna, Galle, Sri Lanka; 2 Duke Global Health Institute, Durham, North Carolina, United States of America; 3 Duke-Ruhuna Collaborative Research Centre, Faculty of Medicine, University of Ruhuna, Galle, Sri Lanka; 4 Department of Microbiology, Faculty of Medicine, Galle, Sri Lanka; 5 Department of Pediatrics, Faculty of Medicine, Galle, Sri Lanka; 6 Institute for Medical Research, Durham, North Carolina, United States of America; 7 Teaching Hospital, Karapitiya, Galle, Sri Lanka; 8 Faculty of Medicine, General Sir John Kotelawala Defence University, Ratmalana, Sri Lanka; 9 Department of Family Medicine and Community Health, Duke University, Durham, North Carolina, United States of America; 10 Department of Medicine, Duke University, Durham, North Carolina, United States of America; Centre hospitalier de Cayenne, FRANCE

## Abstract

**Background:**

Healthcare systems in dengue-endemic countries are often overburdened due to the high number of patients hospitalized according to dengue management guidelines. We systematically evaluated clinical outcomes in a large cohort of patients hospitalized with acute dengue to support triaging of patients to ambulatory versus inpatient management in the future.

**Methods/Principal findings:**

From June 2017- December 2018, we conducted surveillance among children and adults with fever within the prior 7 days who were hospitalized at the largest tertiary-care (1,800 bed) hospital in the Southern Province, Sri Lanka. Patients who developed platelet count ≤100,000/μL (threshold for hospital admission in Sri Lanka) and who met at least two clinical criteria consistent with dengue were eligible for enrollment. We confirmed acute dengue by testing sera collected at enrollment for dengue NS1 antigen or IgM antibodies. We defined primary outcomes as per the 1997 and 2009 World Health Organization (WHO) classification criteria: dengue hemorrhagic fever (DHF; WHO 1997), dengue shock syndrome (DSS; WHO 1997), and severe dengue (WHO 2009). Overall, 1064 patients were confirmed as having acute dengue: 318 (17.4%) by NS1 rapid antigen testing and 746 (40.7%) by IgM antibody testing. Of these 1064 patients, 994 (93.4%) were adults ≥18 years and 704 (66.2%) were male. The majority (56, 80%) of children and more than half of adults (544, 54.7%) developed DHF during hospitalization, while 6 (8.6%) children and 22 (2.2%) adults developed DSS. Overall, 10 (14.3%) children and 113 (11.4%) adults developed severe dengue. A total of 2 (0.2%) patients died during hospitalization.

**Conclusions:**

One-half of patients hospitalized with acute dengue progressed to develop DHF and a very small number developed DSS or severe dengue. Developing an algorithm for triaging patients to ambulatory versus inpatient management should be the future goal to optimize utilization of healthcare resources in dengue-endemic countries.

## Author summary

In countries where dengue is prevalent, hospitals are often overwhelmed due to the high numbers of patient admissions during dengue epidemics. We studied 1064 children and adults hospitalized with acute dengue in Sri Lanka to determine the prevalence of severe disease outcomes to support the development of a system which can limit hospitalizations in the future. We found that only half of patients developed severe disease outcomes during hospitalization and only a small minority of patients developed life-threatening disease. For dengue-prevalent countries, developing systems to identify patients with acute dengue who can be managed without hospital admission should be a priority.

## Introduction

Dengue is considered to be the most important arboviral disease in the world and has the potential to cause life-threatening complications.[1] According to recent estimates, approximately 58–96 million symptomatic dengue infections occur annually, with 10.5 million cases requiring hospitalization.[2,3] Many countries where dengue is considered a public health risk have adopted management guidelines to optimize therapy and to ensure patient safety. The World Health Organization (WHO) developed dengue clinical classification criteria in 1997, and later revised them in 2009, to assist with surveillance, triage, and treatment.[4,5] According to the 2009 WHO guidelines, hospital admission is recommended in the presence of severe dengue; dengue with warning signs that include abdominal pain or tenderness, persistent vomiting, clinical fluid accumulation, mucosal bleeding, lethargy, restlessness, liver enlargement >2 cm, and increase in hematocrit (HCT) concurrent with rapid decrease in platelet count; for those with comorbidities that may make dengue or its management more complicated; and for those with certain social circumstances.[4]

The adoption of guidelines and increased attention to clinical management have been associated with a decrease in dengue mortality to less than 1% in Sri Lanka and many other countries.[6,7] However, healthcare systems in dengue-endemic countries are often overwhelmed during dengue epidemics due to the high number of patients who need monitoring and care in the hospital setting.[3,8]

In Sri Lanka, island-wide annual epidemics of dengue have been occurring since 1989, with all four serotypes of dengue co-circulating in the country.[9] The Ministry of Health in Sri Lanka has developed guidelines for the management of dengue–these build upon the WHO guidelines and provide further country-specific recommendations. The Sri Lankan guidelines recommend inpatient monitoring of patients who have thrombocytopenia (platelet count ≤100,000/μL). In addition, the guidelines recommend that patients with warning signs such as abdominal pain, persistent vomiting, or mucosal bleeding after 3 days of fever or illness be hospitalized for management.[10]

The proportion of patients with acute dengue who develop severe adverse clinical outcomes such as plasma leakage, hemorrhage, and severe dengue has not been prospectively, systematically assessed among hospitalized patients in Sri Lanka. Even in other countries in South Asia with comparable admission criteria, outcomes among hospitalized at-risk dengue cohorts have not been evaluated systematically. With increasing experience and improved outcomes when managing dengue fever in Sri Lanka, the possibility of further refining thresholds for admission and management, while ensuring patient safety, exists. Improved admission criteria become especially important as the demand for hospital beds increases during unexpected viral epidemics. Improved criteria may help triage patients for ambulatory management versus inpatient management, potentially lessening the burden on the healthcare system.[11–14] In order to develop such criteria, a systematic understanding of the spectrum and occurrence of clinical outcomes among patients who meet current criteria for hospital admission is first needed.

## Methods

### Ethics statement

Ethical approval was obtained by the Ethical Review Committee of the Faculty of Medicine, University of Ruhuna (Sri Lanka) and the Institutional Review Board of Duke University (USA). Written informed consent was obtained from patients or their guardians (for patients <18 years of age) and assent was obtained from patients aged 12–17 years.

### Febrile cohort

We conducted a prospective, cohort study of patients admitted to the largest (1,800 bed) tertiary-care hospital in Galle District, Southern Province of Sri Lanka from June 2017 to December 2018. Trained research assistants conducted surveillance among hospitalized children ≥1 year of age and adults with self-reported or documented fever (≥38.0°C/100.4°F) within the prior 7 days. Patients who developed thrombocytopenia (platelet count ≤100,000/μL) within 7 days of admission and who met at least two clinical criteria consistent with dengue were eligible for enrollment. The platelet count threshold of 100,000/μL was selected in accordance with the 2012 Sri Lanka Ministry of Health’s national guidelines for admission among patients with dengue fever.(10) Clinical criteria were extracted from the 2009 World Health Organization (WHO) Case Classification for Probable Dengue or Dengue with Warning Signs and included nausea or vomiting; myalgia or arthralgia; leukopenia (<4,000 x 10^3^ cells/μL); rash or flushing; abdominal pain; clinical or ultrasound evidence of fluid accumulation; bleeding; lethargy or restlessness; liver enlargement; and increase in HCT (≥10% from admission).(4) Patients with focal bacterial infections, fever or thrombocytopenia explained by another etiology as suspected by the primary clinician (*i*.*e*., leukemia), or hospitalization more than 7 days were excluded from the study.

### Study procedures

At enrollment, trained research assistants collected data regarding demographics, chronic medical problems and symptoms, and medications used prior to admission. An acute blood sample was also collected at enrollment from all patients. During hospitalization, research assistants reviewed the patient’s medical records daily to collect information regarding laboratory values and clinical outcomes such as the onset of clinically confirmed plasma leakage, development of shock, and receipt of care in the intensive care unit (ICU). Treatments received by the patient, including blood and platelet transfusions and vasopressors, were also recorded. All patients received routine clinical management by their medical team, and the study team was not involved in any clinical decision-making. Serum was separated and used immediately for rapid NS1 antigen testing if indicated (see Laboratory testing below), with the remainder stored promptly at -80°C for further testing.

### Laboratory testing for acute dengue

Serum rapid NS1 antigen testing (Standard Diagnostics Bioline, Seoul, Korea) was performed for all patients who were enrolled within 5 days of illness onset. NS1 testing was performed on-site at the Department of Microbiology, Faculty of Medicine, University of Ruhuna. Testing was not repeated if a patient had a documented positive NS1 antigen result as an outpatient from a reputed commercial laboratory that was known to use the Standard Diagnostics Bioline rapid NS1 test. Dengue IgM and IgG antibody testing by enzyme-linked immunosorbent assay (ELISA; Standard Diagnostics Bioline, Seoul, Korea) was also performed on-site at the University of Ruhuna. IgM testing was performed on serum of all patients with negative NS1 test results or with duration of illness greater than 5 days. Dengue IgG testing was performed for all enrolled patients.

### Collection of ultrasound examination data

Research assistants recorded results of any ultrasound examinations that were performed and documented in the medical record during the patient’s hospitalization. Per standard hospital protocol for the clinical management of dengue, bedside ultrasound scans of the chest, abdomen, and pelvis are performed by clinicians once the platelet count decreases below 100,000/μL to detect signs of plasma leakage. If the initial scan does not show signs of plasma leakage, scans are typically repeated every 12 hours until the platelet count begins to rise.

### Definitions and outcomes

Patients were classified as having acute dengue if they met the following criteria for laboratory-confirmed dengue: 1) positive rapid NS1 antigen test result if within 5 days of patient’s illness onset, or 2) presence of dengue IgM antibodies if NS1 antigen testing was negative. The presence of IgG in the acute sample differentiated primary from secondary dengue.

Among patients with acute dengue, we assessed for the development of the following primary outcomes: 1) dengue hemorrhagic fever (DHF; 1997 WHO Classification), 2) dengue shock syndrome (DSS; 1997 WHO Classification), and 3) severe dengue (2009 WHO Classification).

DHF was defined as the presence of fever; thrombocytopenia with platelet count <100,000/μL; and evidence of plasma leakage as manifested by 1) ≥20% rise in HCT above average for age and sex, 2) hypoproteinemia <5g/dL, or 3) evidence of pleural effusion or ascites by clinical examination or by ultrasound. As the tourniquet test is rarely performed in routine clinical practice in our setting, we did not include hemorrhagic manifestations as part of the DHF criteria. Including this criterion would have resulted in a large under-estimation of cases. DSS was defined as DHF plus either pulse pressure<20 or hypotension for age (systolic blood pressure <80 if age <5 years or systolic blood pressure <90 if age ≥5 years).

Severe dengue was defined as any of the following: 1) shock with use of vasopressors or pulse pressure <20; 2) fluid accumulation with respiratory distress, defined as clinical or ultrasound evidence of pleural effusions as well as either hypoxia (oxygen saturation <90%) or tachypnea (respiratory rate >20 for patients >5 years of age and respiratory rate >40 for children ≤ 5 years of age); 3) severe bleeding, defined as need for blood transfusions or clinical evidence of severe bleeding such as melaena or haematemesis; or 4) severe organ involvement with serum aspartate aminotransferase (AST) or alanine aminotransferase (ALT) ≥1000U, impaired consciousness with Glasgow Coma Scale score ≤14, cardiac involvement with arrhythmias or heart failure, or acute renal failure requiring dialysis.

Among patients with laboratory-confirmed acute dengue, we also assessed the following secondary outcomes: receipt of crystalloid boluses or colloids; transfusion of blood, platelets, or fresh frozen plasma; receipt of steroids or vasopressors; development of plasma leakage; development of acute renal failure requiring hemodialysis; transfer to the intensive care unit; and death. Plasma leakage was defined as clinical signs of fluid leakage (pleural effusions, ascites) or ultrasound signs of fluid leakage (pleural effusions, ascites, pericholecystic fluid, fluid in the Morrison’s pouch, and pelvic fluid).

### Statistical analysis

We determined the proportion with laboratory-confirmed acute dengue and primary versus secondary dengue. In addition, we determined the proportion who developed our pre-defined primary and secondary outcomes. We performed exploratory analyses to assess associations of age (children versus adult) and type of dengue infection (primary versus secondary) with development of primary outcomes by using the Chi-squared test and the Kruskal-Wallis test as appropriate, with p-value <0.05 being considered statistically significant. All analyses were performed using R version 3.6.3 (Vienna, Austria).

## Results

### Study cohort

Among 1,832 enrolled patients, 1064 (58.1%) were confirmed as having acute dengue: 318 (17.4%) by NS1 rapid antigen testing and 746 (40.7%) by IgM antibody testing. Of patients with positive NS1 antigen test results, 43 (13.5%) tests were performed prior to admission at commercial laboratories and 275 (86.5%) of tests were performed after enrollment at the on-site research laboratory. Among patients with acute dengue, 758 (71.2%) were considered to have secondary dengue based on positive IgG antibody testing of the acute serum sample, with median (IQR) day of illness that the test was performed being 5 (4–6) days. The analyses that follow are limited to the 1064 patients with laboratory-confirmed acute dengue.

### Demographic characteristics and clinical history among patients with acute dengue

Of 1064 patients with acute dengue, 994 (93.4%) were adults ≥18 years and 704 (66.2%) were male (Table 1). Median age among adults with acute dengue was 33.9 years (IQR 25.8–47.6) and median age among children with acute dengue was 14 years (IQR 7.4–17.0). Overall, 177 (16.6%) patients had common chronic medical conditions such as diabetes mellitus (66, 6.2%), hypertension (54, 5.1%), and bronchial asthma (44, 4.1%). Almost all patients (1020, 95.9%) reported use of paracetamol and one-fifth of patients (200,18.8%) reported use of antibiotics in the week prior to admission, with most having used amoxicillin/penicillin (104, 9.8%). Few patients reported the use of other medications such as non-steroidal anti-inflammatory drugs (NSAIDs), steroids, or statins. However, traditional Sinhalese medications such as herbal preparations containing coriander, ginger, and cumin were used commonly in 129 (12.1%).

**Table 1:** Sociodemographic and clinical characteristics of children and adults with acute dengue in Southern Province, Sri Lanka, 2017–2018.

	Children <18 years (n = 70)	Adults ≥18 years (n = 994)	Overall (n = 1064)
	Number (percentage) or median (IQR)	Number (percentage) or median (IQR)	Number (percentage) or median (IQR)
**Sociodemographic characteristics**			
Age (years)	14.0 (7.4–17.0)	33.9 (25.8–47.6)	32.7 (24.3–45.7)
Sex–Male	48 (68.6)	656 (66.0)	704 (66.2)
Residence			
- Urban	13 (18.6)	224 (24.5)	4.2)
- Semi Urban	23 (32.8)	296 (29.8)	0.0)
- Rural	32 (45.7)	422 (42.4)	454 (42.7)
Travel in past 30 days	22 (31.4)	305 (30.7)	0.7)
- Southern Province	10 (14.3)	106 (10.7)	0.9)
- Western Province	12 (17.1)	173 (17.4)	7.4)
- Other province or international	2 (2.8)	51 (5.1)	53 (5.0)
Dengue contact in past 4 weeks	20 (28.6)	299 (30.1)	319 (30.0)
Employed (adults ≥18 years)	---	740 (74.4)	---
**Clinical characteristics**			
Days of fever at admission	4 (3–4.5)	4 (3–5)	4 (3–5)
Days of fever at enrollment	5 (4–6)	5 (3–6)	5 (3–6)
Chronic medical problems	8 (11.4)	169 (17.0)	177 (16.6)
Diabetes mellitus	0	66 (6.7)	66 (6.2)
Hypertension	0	54 (5.4)	54 (5.1)
Bronchial asthma	5 (7.1)	39 (3.9)	44 (4.1)
Ischemic heart disease	0	14 (1.4)	14 (1.3)
**Use of medications prior to admission**			
Paracetamol	67 (95.7)	953 (95.9)	1020 (95.9)
Non-steroidal anti-inflammatory drugs	0	9 (0.9)	9 (0.8)
Steroids	0	9 (0.9)	9 (0.8)
Statins	0	6 (0.6)	6 (0.6)
Antibiotics	7 (10.0)	193 (19.4)	200 (18.8)
Herbal remedies	4 (5.7)	125 (12.6)	129 (12.1)
Papaya leaves^1^	2 (2.8)	13 (1.3)	15 (1.4)
**Clinical symptoms at admission, as reported by patient**			
Nausea/vomiting	39 (55.7)	476 (47.9)	515 (48.4)
Rash	11 (15.7)	89 (9.0)	100 (9.4)
Arthralgia	54 (77.1)	903 (90.8)	957 (89.9)
Myalgia	52 (74.3)	866 (87.1)	918 (86.3)
Fatigue/lethargy	48 (68.6)	835 (84.0)	883 (83.0)
Bleeding	1 (1.4)	60 (6.0)	61 (5.7)
Abdominal pain	30 (42.8)	342 (34.4)	372 (35.0)
Headache	62 (88.6)	877 (88.2)	939 (88.2)
Anorexia	58 (82.8)	873 (87.8)	931 (87.5)
Cough	18 (25.7)	234 (23.5)	252 (23.7)
Sore throat	9 (12.8)	110 (11.1)	119 (11.2)

^1^ A traditional remedy which is believed to have possible benefits in the treatment of acute dengue.

### Clinical and laboratory features in patients with acute dengue

Patients with acute dengue had a median duration of fever of 4 (IQR 3–5) days at admission and 5 days (IQR 3–6) at enrollment (Table 1). Patients commonly had arthralgia (957, 89.9%), headache (939, 88.2%), decreased appetite (931, 87.5%), myalgia (918, 86.3%), and fatigue/lethargy (883, 83.0%) at admission, but some symptoms were significantly more prominent in adults than children: arthralgia (90.8% versus 77.1%), myalgia (87.1% versus 74.3%), and fatigue (84% versus 68.6%). Conversely, children were more likely than adults to report nausea/vomiting (55.7% versus 47.9%), abdominal pain (42.8% versus 34.4%), and rash (15.7% versus 9.0%). Median temperature at admission was 36.8°C (IQR 36.6°C-37.5°C); Table 2). An enlarged liver and tender abdomen were documented in 12 (1.1%) and 138 (13%) of patients, respectively, on physical examination at admission. Rash was present on exam in 79 (7.4%) patients. Similar to reported symptoms, children were more likely to have abdominal tenderness (24.3% versus 12.2%) and rash (15.7% versus 6.8%) on exam than adults.

**Table 2:** Physical examination findings and laboratory results among children and adults with acute dengue in Southern Province, Sri Lanka, 2017–2018.

	Children <18 years (n = 70)	Adults ≥18 years (n = 994)	Overall (n = 1064)
	Number (percentage) or median (IQR)	Number (percentage) or median (IQR)	Number (percentage) or median (IQR)
**Physical examination findings at admission**			
Temperature (°C)	36.6 (36.6–37.7)	36.8 (36.6–37.5)	36.8 (36.6–37.5)
Median systolic blood pressure	106.5 (100.0–113.5)	113.0 (108.8–122.0)	112.0 (107.0–120.0)
Median diastolic blood pressure	70.0 (60.0–70.0)	74.0 (70.0–80.0)	72.0 (70.0–80.0)
Heart rate	88 (80–98)	80 (72–88)	80 (72–88)
Jaundice	0	6 (0.6)	6 (0.5)
Chest dullness	5 (7.1)	74 (7.4)	79 (7.4)
Enlarged liver	1 (1.4)	11 (1.1)	12 (1.1)
Tender abdomen	17 (24.3)	121 (12.2)	138 (13.0)
Ascites	5 (7.1)	99 (10.0)	104 (9.8)
Rash	11 (15.7)	68 (6.8)	79 (7.4)
Central nervous system abnormalities	2 (2.8)	22 (2.2)	24 (2.2)
Capillary refill ≥2 sec	3 (4.3)	21 (2.1)	24 (2.2)
**Laboratory findings**			
White blood cell count at admission (x10^3^ cells/uL)	3.63 (2.76–6.04)	3.90 (2.96–5.83)	3.90 (2.95–5.84)
Platelet count at admission (x10^3^/uL)	93,500 (58,000–122,500)	81,000 (38,000–114,000)	82,000 (39,500–114,500)
Nadir white blood cell count (x10^3^ cells/uL)	2.75 (2.06–4.29)	2.91 (2.18–4.23)	2.90 (2.16–4.24)
Nadir platelet count (x10^3^/uL)	33,500 (16,250–56,750)	21,000 (8,000–50,000)	21,500 (8,000–51,000)
Transaminitis (AST or ALT >120U) at admission^1^	25 (35.7)	403 (40.5)	428 (40.2)

At admission, median white blood cell count was 3.90 x 10^3^ cells/μL (IQR 2.95–5.84) and median platelet count was 82 x 10^6^/ μL (IQR 39.5–114.5). During hospitalization, nadir white blood cell count was 2.90 x 10^3^ cells/μL (IQR 2.16–4.24) and nadir platelet count was 21.5 x 10^6^/μL (IQR 8–51). A large proportion (428, 40.2%) of patients had transaminitis (AST/ALT >120U) at admission. Nadir platelet count was significantly lower in adults than in children (21,000 x10^3^/uL versus 33,500 x10^3^/uL).

### Primary outcomes (WHO case classification outcomes)

The majority (56, 80%) of children and over half of adults (544, 54.7%) with acute dengue developed DHF during hospitalization, while only 6 (8.6%) of children and 22 (2.2%) of adults developed DSS, according to the 1997 WHO classification criteria (Table 3). Overall, 10 (14.3%) of children and 113 (11.4%) of adults developed severe dengue according to the 2009 WHO classification criteria. We performed a sensitivity analysis limiting analysis to patients with platelet count <100,000 cells/ul at admission, and found a similar distribution of outcomes: among children (n = 32), 78.1% developed DHF, 6.2% developed DSS, and 15.6% developed severe dengue while among adults (n = 593), 61.9% developed DHF, 2.4% developed DSS, and 13.6% developed severe dengue. Primary versus secondary dengue was not associated with the development of DHF, DSS, or severe dengue in children (Table 4), while DHF was more common in secondary dengue versus primary dengue in adults (60.8% versus 38.8%, p < .001).

**Table 3:** Primary clinical outcomes, as defined by the 1997 and 2009 World Health Organization classification criteria for dengue, among children and adults with acute dengue in Southern Province, Sri Lanka, 2017–2018.

	No (%) in children (n = 70)	No (%) in adults (n = 994)	No (%) overall (n = 1064)	p-value
Dengue hemorrhagic fever	56 (80.0)	544 (54.7)	600 (56.4)	< .001
-Fever	70 (100)	994 (100)	1064 (100)	---
-Thrombocytopenia <100,000 cells/ul	70 (100)	994 (100)	1064 (100)	---
-Evidence of plasma leakage^1^	56 (80.0)	544 (54.7)	600 (56.4)	< .001
-HCT rise of ≥20%^2^	54 (77.1)	275 (27.7)	329 (30.9)	< .001
-Clinical or ultrasonographic evidence of plasma leakage	27 (38.6)	432 (43.5)	459 (43.1)	0.565
-Hypoproteinemia	1 (1.4)	11 (1.1)	12 (1.1)	1.000
Dengue shock syndrome^3^	6 (8.6)	22 (2.2)	28 (2.6)	0.005
-Pulse pressure <20	1 (1.4)	21 (2.1)	22 (2.1)	1.000
-Hypotension for age	8 (11.4)	25 (2.5)	33 (3.1)	< .001
Severe dengue^4^	10 (14.3)	113 (11.4)	123 (11.6)	0.586
-Shock^5^	1 (1.4)	26 (2.6)	27 (2.5)	0.828
-Fluid accumulation with respiratory distress^6^	7 (10.0)	62 (6.2)	69 (6.5)	0.325
-Severe bleeding^7^	2 (2.8)	23 (2.3)	25 (2.3)	1.000
-Severe organ involvement^8^	2 (2.8)	25 (2.5)	27 (2.5)	1.000

^1^Evidence of plasma leakage includes both clinical and ultrasound plasma leakage.

^2^HCT (hematocrit) standard values were used: 36% for adult females, 40% for adult males, and 35% for children.

^3^Dengue shock syndrome was defined as dengue hemorrhagic fever plus either pulse pressure<20 or hypotension for age (systolic blood pressure <80 if age <5 years or systolic blood pressure <90 if age ≥5 years).

^4^Severe dengue was defined as fever plus shock, fluid accumulation with respiratory distress, severe bleeding, or severe organ involvement.

^5^Shock was defined as pulse pressure < 20 or the use of vasopressors.

^6^Fluid accumulation with respiratory distress was defined as clinical or ultrasound evidence of pleural effusions as well as either hypoxia (oxygen saturation <90%) or tachypnea (respiratory rate >20 for patients >5 years of age and respiratory rate >40 for children ≤ 5 years of age.

^7^Severe bleeding was defined as need for blood transfusions or clinical evidence of severe bleeding such as melaena or haematemesis.

^8^ Severe organ involvement was defined as AST/ALT ≥1000 IU/L, Glasgow coma score ≤14, cardiac involvement with arrhythmias or heart failure, or acute renal failure requiring dialysis

**Table 4:** Primary clinical outcomes among children and adults with primary versus secondary dengue, Southern Province, Sri Lanka, 2017–2018.

	Primary dengue	Secondary dengue	Overall	p-value
**Children**	Number (%), n = 28	Number (%), n = 41	Number (%), n = 69	
Dengue hemorrhagic fever	22 (78.6)	34 (82.9)	56 (81.2)	0.888
Dengue shock syndrome	1 (3.6)	5 (12.2)	6 (8.7)	0.416
Severe dengue	2 (7.1)	8 (19.5)	10 (14.5)	0.278
**Adults**	Number (%), n = 273	Number (%), n = 717	Number (%), n = 990	
Dengue hemorrhagic fever	106 (38.8)	436 (60.8)	542 (54.7)	<0.001
Dengue shock syndrome	5 (1.8)	17 (2.4)	22 (2.2)	0.784
Severe dengue	34 (12.4)	78 (10.9)	112 (11.3)	0.557

### Secondary outcomes

#### Plasma leakage in acute dengue

During hospitalization, 197 (18.5%) of patients developed clinical signs of fluid leakage, defined as pleural effusions (194, 18.2%) or ascites (29, 2.7%) on physical examination. Median day of illness during which patients showed clinical signs of pleural effusion or ascites were both 6 days (IQR 5–7). Ultrasonography to detect plasma leakage was performed on 428 (40.2%) patients at least once during hospitalization. Overall, 120 (11.3%) patients with acute dengue developed significant plasma leakage in the form of pleural effusions (105, 9.9%) or ascites (32, 3.0%), as detected by ultrasound. Median onset day of both pleural effusions and ascites was day 6 of illness (IQR 5–7 for both). Less clinically significant signs of plasma leakage were also noted by ultrasound: 110 (10.3%) with pericholecystic fluid detected at day 6 (IQR 5–7 days), 244 (22.9%) with fluid in Morrison’s pouch detected at day 6 (IQR 5–7 days), and 42 (3.9%) with pelvic fluid detected at day 6 (IQR 5–7 days).

#### Treatments received and hospitalization outcomes

All patients received normal saline infusions during admission, in accordance with the Sri Lanka national guidelines for dengue management.(10) Few patients (62, 5.8%) received colloids, blood transfusions (18, 1.7%), platelet transfusions (5, 0.5%), or fresh frozen plasma (4, 0.4%; Table 5). One-fifth of patients received tranexamic acid while <1% received treatment with vasopressors or steroids. A total of 17 (1.6%) patients were transferred to the intensive care unit for management. Median duration of hospitalization was 4 (IQR 3–5) days and 2 (0.2%) patients died during hospitalization, with one dying from multi-organ failure and the other dying from rapid plasma leakage and shock.

**Table 5:** Secondary outcomes during hospitalization among children and adults with acute dengue in Southern Province, Sri Lanka, 2017–2018.

	Children <18 years (n = 70)	Adults ≥18 years (n = 994)	Overall (n = 1064)
	Number (percentage) or median (IQR)	Number (percentage) or median (IQR)	Number (percentage) or median (IQR)
Crystalloid bolus use	23 (32.8)	425 (42.8)	448 (42.1)
Colloid use	3 (4.3)	59 (5.9)	62 (5.8)
Blood transfusion	1 (1.4)	17 (1.7)	18 (1.7)
Platelet transfusion	0	5 (0.5)	5 (0.5)
Fresh frozen plasma transfusion	0	4 (0.4)	4 (0.4)
Tranexamic use	8 (11.4)	201 (20.2)	209 (19.6)
Steroid use	2 (2.8)	4 (0.4)	6 (0.6)
Vasopressor use	0	5 (0.5)	5 (0.5)
Acute renal failure requiring dialysis	0	4 (0.4)	4 (0.4)
Intensive care unit transfer	1 (1.4)	16 (1.6)	17 (1.6)
Hospitalization duration (days)	4 (3–5)	4 (3–5)	4 (3–5)
Death	0	2 (0.2)	2 (0.2)

^1^AST is aspartate transaminase and ALT is alanine transaminase.

## Discussion

In this large, prospective cohort of patients hospitalized with acute dengue in the Southern Province of Sri Lanka, one-half progressed to develop clinical signs of plasma leakage and DHF. Notably, among patients in our study who progressed to DHF, only a small number developed DSS or severe dengue, likely reflecting prompt management by clinicians in accordance with national guidelines. Further, few patients received ICU care, blood transfusions, or platelet transfusions. Despite all patients in our study meeting criteria for hospital admission as per the national guidelines in Sri Lanka, our results suggest that a substantial proportion of patients may not require intensive management in the inpatient setting. Identifying robust predictors of dengue severity and developing an algorithm for safely triaging patients to ambulatory versus intensive inpatient management may thus be of great benefit to the country and other dengue-endemic areas worldwide, where the public healthcare system can become overwhelmed during peak dengue season.

Surprisingly, few prospective studies have been conducted with the explicit purpose of quantifying clinical outcomes in patients with acute dengue in high-burden settings. Most data regarding clinical outcomes have been gathered from retrospective cohorts, which are subject to limitations in terms of data quality and breadth. In our study, we found differences in the proportion of children versus adults who developed severe outcomes: 80% of children versus 54.5% of adults developed DHF, with more plasma leakage in children versus adults, and children were also more likely to develop DSS and severe dengue. Other cohorts of dengue in the recent past have reported variable proportions progressing to DSS, severe dengue, or mortality, and some have explored differences between children and adults. A large multi-year, retrospective study evaluating over 17,000 Saudi Arabian patients with acute dengue reported a lower proportion of children versus adults developing severe dengue (1.2% versus 2.4%, respectively), as well as lower mortality rates in children versus adults with severe dengue (8.9% versus 10.7%, respectively).[15] Overall mortality in the cohort was 0.14%. In another cross-sectional, retrospective cohort in Brazil of over 5000 individuals, 7.6% of adults versus 3.5% of children developed severe dengue.[16] However, in a prospective study in Nicaragua of over 3,000 patients, the authors found that children were more likely to develop severe disease than adults.[17] Plasma leakage was detected in 40% of infants, 30% of children, and 15% of adults, and clinical shock was detected in 40% of infants, 35% of children, and 12% of adults. Finally, in a five-year prospective cohort study including 496 children in Jakarta Indonesia, 32% were classified as DHF, 31% were classified as DSS, and a significant proportion (28%) of patients with dengue fever patients progressed to DHF or DSS, but there were no deaths.[18] The significant differences in tendency for plasma leakage and the likelihood of developing DHF and DSS, based on age, suggest the need for developing separate triaging algorithms for children and adults. It is important to note that a significant proportion of hospitalized patients never progressed to DHF, and identifying predictors to triage this group of patients will be important for limiting hospital admissions.

In our dengue cohort, overall mortality was 0.2%, with the two deaths seen only among the 994 adult patients. One patient died from multi-organ failure and one patient died from rapid fluid leakage and shock. Bedside ultrasound to detect plasma leakage was performed and documented as part of routine care in 40% of the cohort. Clinical evidence of plasma leakage was detected only in 18% of the cohort, but ultrasound improved detection of plasma leakage to 43%, with subsequent increased categorization of patients to DHF. Early detection of fluid leakage by bedside ultrasound and appropriate fluid management according to the guidelines issued by the Sri Lanka Ministry of Health is likely to have made a significant improvement in dengue mortality in recent years, as also seen in other countries.

Worldwide, researchers have attempted to identify predictors of DHF and severe dengue to develop criteria to triage patients for safe outpatient care. A prospective cohort study carried out in Singapore with 499 cases of confirmed dengue suggested that ambulatory care with monitoring, even in the presence of warning signs, was safe.[14] In this study, the use of admission criteria plus clinical judgement significantly reduced hospital admission compared to the use of warning signs alone as hospital admission criteria. The outcomes of patients with warning signs who were managed as outpatients were similar to those who were managed as inpatients. Several retrospective and prospective studies have reported the value of specific clinical and early laboratory parameters to identify patients at risk of progressing to severe dengue.[19–24] Among the clinical predictors, many studies report age, presence of comorbidities, nutritional status, and the presence of specific clinical features on admission as predictive of DHF or severe dengue.[19–24] In addition, simple laboratory parameters also have been shown to be predictive of severe disease.[19,21,24] More recently, sophisticated biomarkers such as chymase and predictive gene sets have also been shown to identify patients who may progress to developing severe dengue.[25,26] Identifying such predictors of dengue severity and developing separate algorithms for adults and children for triaging patients to ambulatory versus inpatient management should be the future goal to optimize utilization of the healthcare systems in dengue-endemic countries.

Some limitations must be noted. Given resources available, we were not able to obtain convalescent specimens from patients and may have missed some cases of laboratory-confirmed acute dengue. In addition, since patients were enrolled anytime within 7 days of admission, patients with primary dengue may have started to develop IgG antibodies by enrollment and may have been misclassified as having secondary dengue. However, our findings from a 2012–2013 cohort study in the same region which included comprehensive testing of both acute and convalescent sera indicated that 63.1% of patients had secondary dengue.[27] This proportion would be expected to increase over time give the increased circulation of dengue in Sri Lanka in recent years, thus the 71.2% seen in the current study is within expected estimates. For enrollment, we chose to limit our analyses patients who developed thrombocytopenia to reduce variability compared to subjective clinical signs, and to increase generalizability to other settings, since thrombocytopenia is a common admission criterion and a component of the DHF definition. Including other patients would only be expected to decrease the proportion who develop severe disease and who require inpatient management. Ultrasound was only performed and documented for 40% of patients with acute dengue. It is possible that ultrasound was performed on other patients and not documented, leading to a potential under-estimation of severity in our cohort. However, it is likely that documentation was more frequently omitted in patients who had clinically insignificant plasma leakage as opposed to patients who had severe plasma leakage. Finally, we enrolled patients who developed thrombocytopenia at any time during the first 7 days of admission. It is possible that early, inpatient clinical management may have decreased severe outcomes in these patients who may have otherwise progressed to more severe disease in an outpatient setting. However, our secondary analysis limited to patients who were thrombocytopenic on admission showed similar proportions who developed DHF, DSS, and severe dengue.

In conclusion, in this large, prospective cohort of patients hospitalized with acute dengue and the potential to develop severe disease, only one half progressed to DHF and only a very small number developed DSS or severe dengue. Our findings suggest potential for triaging patients with acute dengue to ambulatory care versus in-hospital management. Identifying predictors of severe disease and developing separate triaging algorithms for children and adults should be our future goal to optimize utilization of healthcare systems in dengue-endemic countries.
