# Correction: Outcomes among children and adults at risk of severe dengue in Sri Lanka: Opportunity for outpatient case management in countries with high disease burden

**DOI:** 10.1371/journal.pntd.0010498

**Published:** 2022-05-31

**Authors:** Champica K. Bodinayake, Ajith DeS Nagahawatte, Vasantha Devasiri, Niroshana J. Dahanayake, Gaya B. Wijayaratne, Nayani P. Weerasinghe, Madureka Premamali, Tianchen Sheng, Bradley P. Nicholson, Harshanie A. Ubeysekera, Ruvini MP Kurukulasooriya, Aruna D. de Silva, Truls Østbye, Christopher W. Woods, L. Gayani Tillekeratne

The ninth author’s name is spelled incorrectly. The correct name is: Bradley P. Nicholson.
